# Changes of absorbed dose rate in air in metropolitan Tokyo relating to radiocesium released from the Fukushima Daiichi Nuclear Power Plant accident: Results of a five-year study

**DOI:** 10.1371/journal.pone.0224449

**Published:** 2019-10-24

**Authors:** Kazumasa Inoue, Hiroshi Tsuruoka, Hideo Shimizu, Moeko Arai, Nimelan Veerasamy, Mizuho Tsukada, Ken Ichimura, Shuto Nakazawa, Yoshiaki Taguchi, Masahiro Fukushi

**Affiliations:** 1 Department of Radiological Sciences, Graduate School of Human Health Sciences, Tokyo Metropolitan University, Arakawa-ku, Tokyo, Japan; 2 Department of Radiological Sciences, Tsukuba International University, Tsuchiura, Ibaraki, Japan; Fukushima Medical University School of Medicine, JAPAN

## Abstract

Car-borne surveys were carried out in metropolitan Tokyo, Japan, in 2015, 2016, 2017 and 2018 to estimate the transition of absorbed dose rate in air from the Fukushima Daiichi Nuclear Power Plant accident. Additionally, the future transition of absorbed dose rates in air based on this five-year study and including previously reported measurements done in 2014 by the authors was analyzed because central Tokyo has large areas covered with asphalt and concrete. The average absorbed dose rate in air (range) in the whole area of Tokyo measured in 2018 was 59 ± 9 nGy h^-1^ (28–105 nGy h^-1^), and it was slightly decreased compared to the previously reported value measured in 2011 (61 nGy h^-1^; 30–200 nGy h^-1^). In the detailed dose rate distribution map, while areas of higher dose rates exceeding 70 nGy h^-1^ had been observed on the eastern and western ends of Tokyo after 2014, the dose rates in these areas have decreased yearly. Especially, the decreasing dose rate from radiocesium (Cs-134 + Cs-137) in the eastern end of Tokyo which is mainly covered by asphalt was higher than that measured in the western end which is mainly covered by forest. The percent reductions for the eastern end in the years 2014–2015, 2015–2016, 2016–2017 and 2017–2018 were 49%, 21%, 18% and 16%, and those percent reductions for western end were 26%, 18%, 6% and 3%, respectively. Additionally, the decrease for dose rate from radiocesium depended on the types of asphalt, and that on porous asphalt was larger than the decrease on standard asphalt.

## Introduction

The distribution of environmental radiation in eastern Japan was dramatically changed after the Fukushima Daiichi Nuclear Power Plant (F1-NPP) accident that occurred in March 2011 [1]. This accident was a level 7 nuclear accident on the International Nuclear and Radiological Event Scale. During the accident, large amounts of fission products were released into the atmosphere and the Pacific Ocean. The released total amounts of artificial radionuclides were estimated to be 100–500 PBq of ^131^I and 6–20 PBq of ^137^Cs and they are about 10% and 20% of the respectively estimated amounts emitted in the Chernobyl accident [2]. Eight years after the F1-NPP accident, Cs-134 (half-life 2.06 y) and Cs-137 (half-life 30.17 y) remain as the major concern from a radiological safety viewpoint.

**Fig 1:**
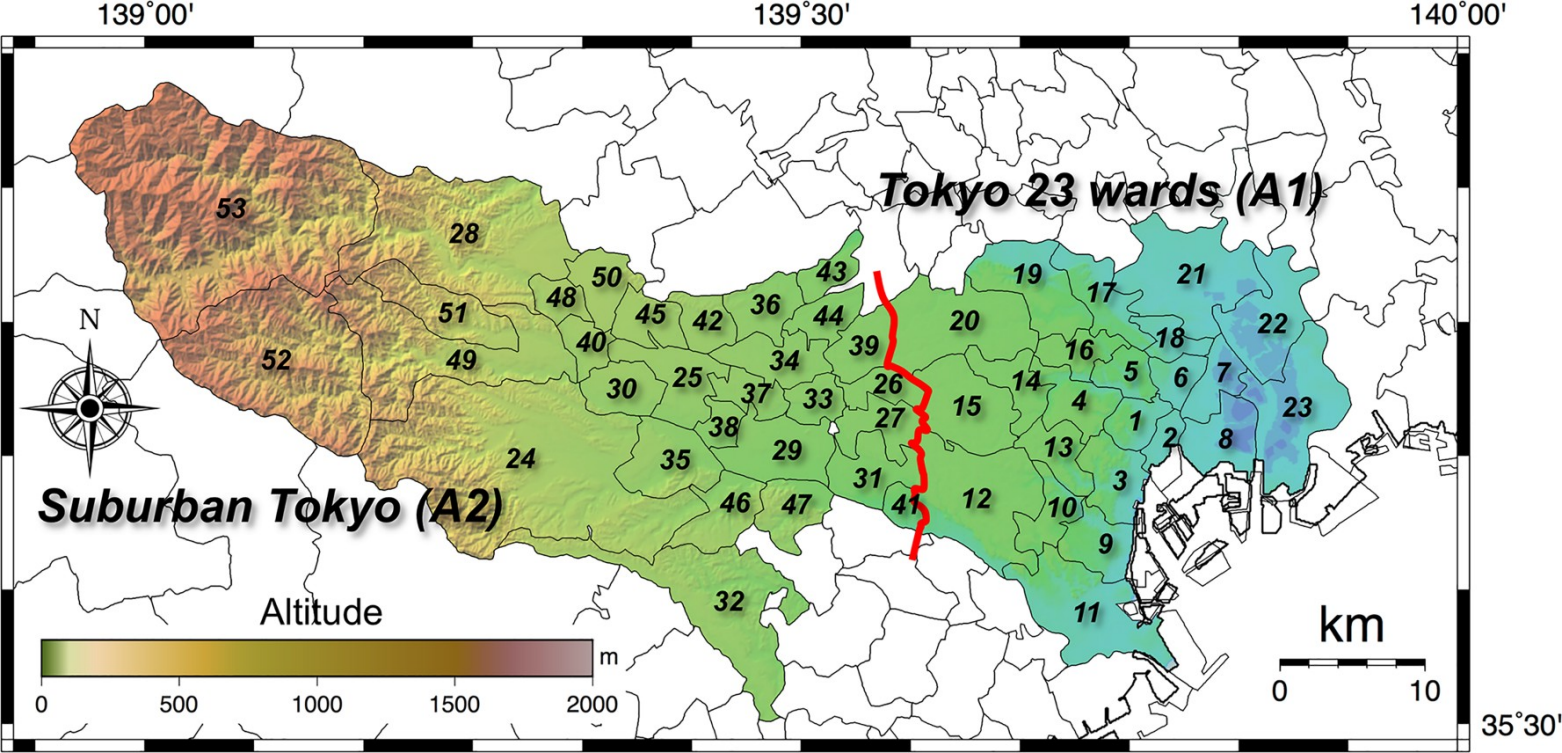
Location of Tokyo municipalities consisting of 23 wards (A1) and 30 cities, towns and villages (A2). The number for each administrative district (#1 - #53) is an ID number that is given in this paper by reference to the Japanese Industrial Standards. The color scale gives the altitudes within the districts. This map was drawn using the GMT [15] and GSI maps of the Geospatial Information Authority of Japan [16].

In metropolitan Tokyo, 220 km southwest of the F1-NPP, such artificial radionuclides as I-131, Cs-134 and Cs-137 were wet-deposited on March 21–23, 2011 by rainfalls [3, 4]. According to the Tokyo Metropolitan Government, the average absorbed dose rate in air (range) that was observed in June 2011 was 61 ± 24 nGy h^-1^ (30–200 nGy h^-1^) based on the measurements at 100 locations in Tokyo [5]. The authors measured and reported detailed data in 2003 for absorbed dose rates in air (*n* = 669) for Tokyo that were obtained based on a car-borne survey using a 2-in × 2-in NaI(Tl) scintillation spectrometer (excluding the Pacific Ocean islands that are within the Tokyo Government’s jurisdiction) [6] and found that the average absorbed dose rate in air was 49 ± 6 nGy h^-1^ (Fig 1). The area with the highest dose rate due to radionuclides deposited from the F1-NPP accident was Katsushika Ward which is located in the northeastern part of Tokyo (#22 in Fig 1). The absorbed dose rate in air measured in July 2011 in Katsushika Ward was 268 nGy h^-1^, which was 6.9 times higher than the value before the accident (i.e., 39 nGy h^-1^) [7].

The distribution of environmental radiation for metropolitan Tokyo was observed by air-borne monitoring after the F1-NPP accident in September 2011 and May 2012 by the Nuclear Regulation Authority, Japan [8]. However, the observed dose rates for Tokyo were below 0.1 μSv h^–1^ almost everywhere, meaning that a detailed discussion of contamination in the metropolitan area was not possible. The authors carried out measurements in 2014 and made a detailed dose rate distribution map for the whole area of Tokyo. Higher dose rate areas exceeding 70 nGy h^-1^ were observed on the eastern and western ends of Tokyo [5].

The continuous monitoring for environmental radiation is required to understand the diffusion, deposition, migration and situation of released artificial radionuclides, and to assess external and internal exposure doses for residents from just after the accident and in the future. Some researchers have reported the reduction of dose rate or radioactivity concentration after the Chernobyl and F1-NPP accidents [7, 9–13]. In metropolitan Tokyo, the dose rates have been continually observed at eight monitoring posts [14]. However, these “point” data are not sufficient to obtain a complete view of the absorbed dose rate in air in Tokyo and to serve the above purposes. Especially, there have been no reports about the continually observed reduction of absorbed dose rates in air measured in a big metropolitan center such as Tokyo which has large areas covered with asphalt and concrete, and it is expected that there is a difference in the reduction of absorbed dose rates compared to that of the countryside which is mainly covered with forests or is bare ground.

In this paper, car-borne surveys were carried out for the whole metropolitan Tokyo area in November of the four years 2015, 2016, 2017 and 2018. The changes of impact on absorbed dose rates relating to the F1-NPP accident were observed by including data measured in December 2014 [5], and the future transition of absorbed dose rates in air was analyzed based on this five-year study.

## Materials and methods

The fixed-point observations were carried out on private land after obtaining specific permission from the land owners. The field studies did not involve endangered or protected species.

### Survey area

**Fig 2:**
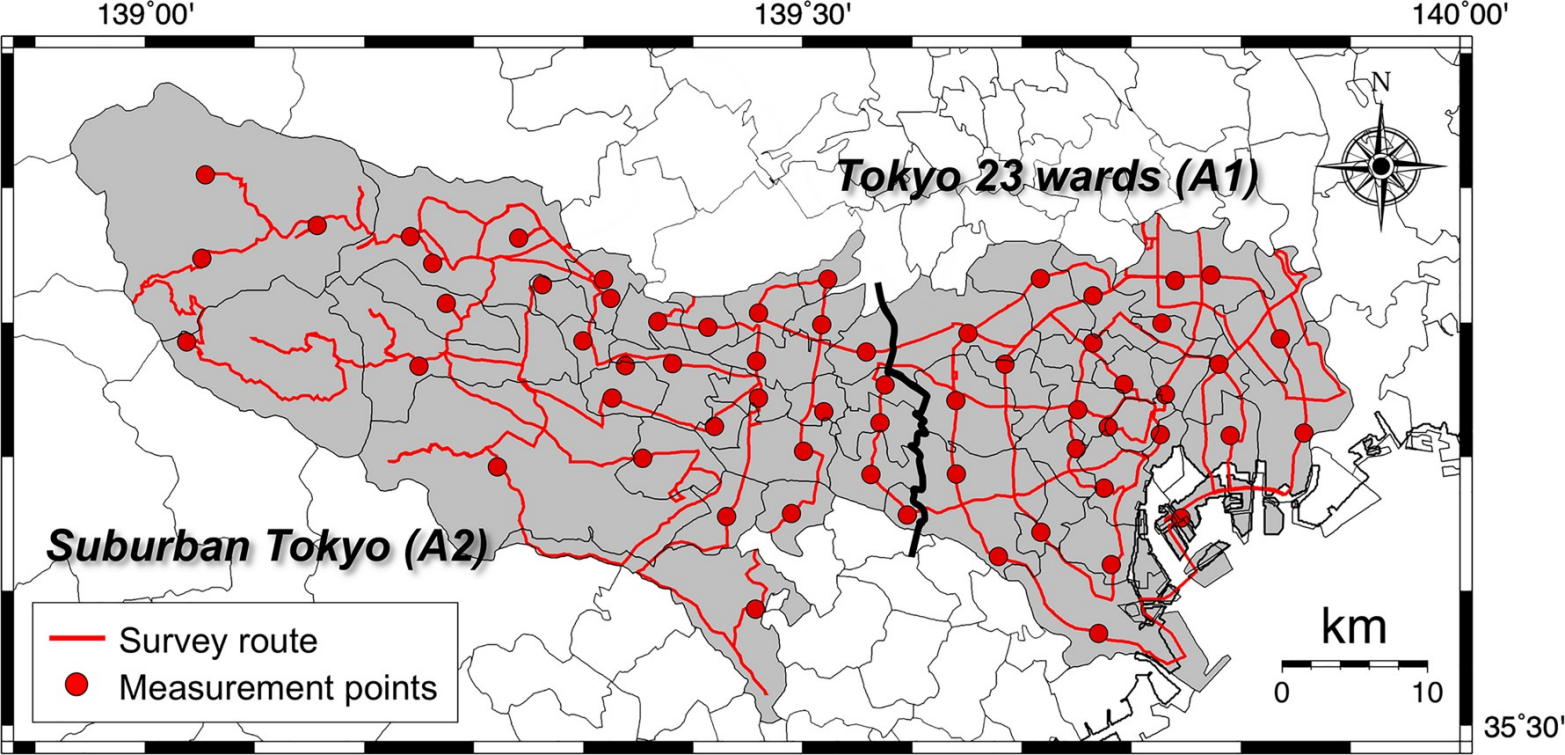
The survey routes for measuring the count rates in metropolitan Tokyo. Car-borne surveys were carried out using a 3-in × 3-in NaI(Tl) scintillation spectrometer in November of the four years, 2015, 2016, 2017 and 2018. Total distances traveled were 725 km for each year. The circles represent the locations where fixed-point measurements were made outside the car (*n* = 61).

The measurements of the count rates were carried out during November of the four years 2015, 2016, 2017 and 2018, in Tokyo, excluding its Pacific island chain that lies in a southeastern direction from the main Japanese island. The survey route encompassed the 23 wards (A1 in Fig 1) and suburban Tokyo (30 cities, towns and villages; A2 in Fig 1). Main roads excluding expressways were used to the extent possible, and selection was primarily centered on residential areas (Fig 2). The survey route had a total distance of 725 km. The weather was sunny or cloudy throughout the measurement days. The route map was drawn using the Generic Mapping Tools (GMT) [15] and GSI maps of the Geospatial Information Authority of Japan [16].

### Car-borne survey

A car-borne survey technique is very useful to make a fast assessment of the dose rate in a large area [17]. In this study, car-borne surveys were carried out over asphalt pavements using a 3-in × 3-in NaI(Tl) scintillation spectrometer with a global positioning system (EMF-211, EMF Japan Co., Osaka, Japan). This survey system combined the NaI(Tl) scintillation spectrometer and a multi-channel analyzer (GAMMA-RAD5, AMPTEK, Bedford, MA, USA). This was the same system as had been used by the authors in 2014 [5] and all procedures were the same in every survey. The NaI(Tl) scintillation spectrometer was positioned 1 m above the ground surface at the center of the car (Aqua for 2015 and Prius for 2016–2018, Toyota, Japan). Measurement of count rate inside the car was performed every 30 s while the car was moving with a speed around 35 km h^-1^. The car windows were kept closed during those measurements. Latitude and longitude at each measurement point were measured at the same time as the count rates. The count rates within gamma-ray energies of 50 keV– 3.2 MeV were recorded. The photon peaks of K-40 (E_*γ*_ = 1.464 MeV) and Tl-208 (E_*γ*_ = 2.615 MeV) were used for gamma-ray energy calibration from the channel number and gamma-ray energy before the measurements. The peak position was determined accurately by smoothing the gamma-ray pulse height distribution. Because count rates were measured inside the car, shielding by the car body was also estimated by making measurements inside and outside the car at 30-s intervals during 2 min at 61 locations (circles in Fig 2). Those measurements were done above asphalt surfaces because so much of the metropolitan area has been extensively covered with asphalt and concrete, making it impossible to find open fields near roadsides for the measurement. The distance from the car was at least 5–15 m for the measurement depending on the situation. The shielding factor (*SF*) was calculated from the correlation between count rates inside and outside the car. In this study, *SF* was obtained from the slope of the regression line in the relation between inside and outside count rates. The count rates inside the car was then multiplied with this *SF*.

The gamma-ray pulse height distributions were also measured outside the car for 10 min, at 61 locations (circles in Fig 2) for estimating the dose rate conversion factor (*DCF*) (nGy h^-1^/cps). The gamma-ray pulse height distributions were then unfolded using the 22 × 22 response matrix method [18] and absorbed dose rates in air were calculated. These calculated dose rates were used to estimate *DCF* as the correlation between dose rates and count rates outside the car because it is difficult to obtain the photon peaks in the 30-s measurement of the car-borne survey [19]. In this study, the dose conversion factor was obtained from the slope of the regression line in the relation between corrected outside count rates and absorbed dose rates in air obtained at 61 locations. The obtained *DCF* was multiplied by the corrected count rates outside the car, and the absorbed dose rates (nGy h^-1^) were calculated. Thus, the absorbed dose rate in air outside the car at 1 m above the ground surface (*D_air_*) can be calculated using the following equation:

$$D_{air}=C_{in}\times SF\times DCF$$ (1)

where *C_in_* is the count rate (cps) inside the car obtained by the measurements for every 30-s interval. All obtained data from the car-borne surveys were plotted on the distribution of absorbed dose rates in air in Tokyo using a minimum curvature algorithm of GMT [15]. This is the method for interpolating data by presuming a smooth curved surface from the data of individual points.

For more detailed analysis, the calculated absorbed dose rates in air obtained at 61 locations (Fig 2) were separated as artificial radionuclides (Cs-134 and Cs-137) and natural radionuclides (K-40, U-238 series and Th-232 series) using the 22 × 22 response matrix method to assess the changes of impact on dose rate from the F1-NPP accident and the seasonal variation of dose rate from natural radionuclides. The obtained gamma-ray pulse height distribution was converted to the energy bin spectrum of incident gamma-rays which is a distribution of gamma-ray flux density to each energy bin. The energy intervals for the bins were given from the literature [18]. The calculation for the 22 × 22 response matrix for the 3-in × 3-in NaI(Tl) scintillation spectrometer was done using the Monte Carlo code, SPHERIX [20]. The gamma-ray flux density and dose rate per unit solid angle were assumed to be almost isotropic in a natural environment. In the location that has a mixture of artificial and natural radionuclides, it is necessary to remove the interference of higher energy peaks from each energy bin for each radionuclide. In this study, the distribution ratio of gamma-ray flux from artificial and natural radionuclides to each energy bin was estimated using the same Monte Carlo code and the interference with each radionuclide was removed. A total of one million histories were traced for each natural radionuclide. After unfolding the gamma-ray pulse height distribution, clear peaks from Cs-134 (energy range: 0.55–0.65 MeV and 0.75–0.85 MeV), Cs-137 (energy range: 0.65–0.75 MeV), K-40 (energy range: 1.39–1.54 MeV), Bi-214 (energy range: 1.69–1.84 MeV and 2.10–2.31 MeV) and Tl-208 (energy range: 2.51–2.27 MeV) were observed in the spectrum. The absorbed dose rates in air from natural and artificial radionuclides could be separated using those techniques. The details of the analysis using the 22 × 22 response matrix method have been described previously [5, 18].

## Results and discussion

### Shielding and dose conversion factors

The *SF*s values for each measurement year were obtained to calculate absorbed dose rates in air as shown in Table 1. The *SF*s ranged from 1.35 to 1.56. The *SF* is influenced by the type of car used in a survey, the number of passengers and the scintillation spectrometer position inside the car. In previous reports, *SF*s have ranged from 1.1 to 1.9 [5–7, 17, 19, 21–25], and the presently obtained *SF*s were in this range. The coefficient of determination (*R*^2^) from measurement correlations ranged from 0.661 to 0.774 and the calculated *R*^2^ for metropolitan Tokyo had lower values compared to those measured in another Japanese report (*R*^2^ = 0.967, *n* = 35) [23]. In the measurement of count rates outside the car, a scintillation spectrometer ideally should be placed in an open space at a distance of 10–20 m from the car and nearby artificial structures to eliminate the impact on count rates outside the car from these structures. The population density of metropolitan Tokyo is ranked first in the world and there are many artificial structures. Therefore, measurements at such an ideal place were impossible, and that resulted in mid-level correlations being exhibited by the metropolitan Tokyo measurements.

The *DCF*s were then obtained from the correlation between count rate outside the car and absorbed dose rate in air calculated from software using the 22 × 22 response matrix method [18] (Table 1). The *DCF* (nGy h^-1^/cps) was evaluated as 0.16. In the method for determining the ambient dose rate from count rate, the dispersion of dose conversion coefficient is affected by the abundance ratio of K-40, U-238, Th-232, Cs-134 and Cs-137. Thus, this effect resulted in a negligible change in this study. The obtained *R*^2^ from measurements ranged from 0.728 to 0.876. The dispersion of *DCF* is affected by the abundance ratios of K-40, U-238 series, Th-232 series [26], Cs-134 and Cs-137. Lower *R*^2^ values were exhibited in the Tokyo metropolitan area measurements compared to those for other areas in Japan [23].

**Table 1:** Calculated shielding factors *SF*s and dose conversion factors *DCF*s.

Year	*n*^a^	SF	Standard uncertainty of SF	DCF (nGy h^-1^/cps)	Standard uncertainty of DCF
2015	61	1.35	0.08	0.16	0.01
2016	61	1.56	0.08	0.16	0.01
2017	61	1.52	0.07	0.16	0.01
2018	61	1.52	0.08	0.16	0.01

^a^ Number of measurements

### Changes of distribution of absorbed dose rates in air in metropolitan Tokyo

**Fig 3:**
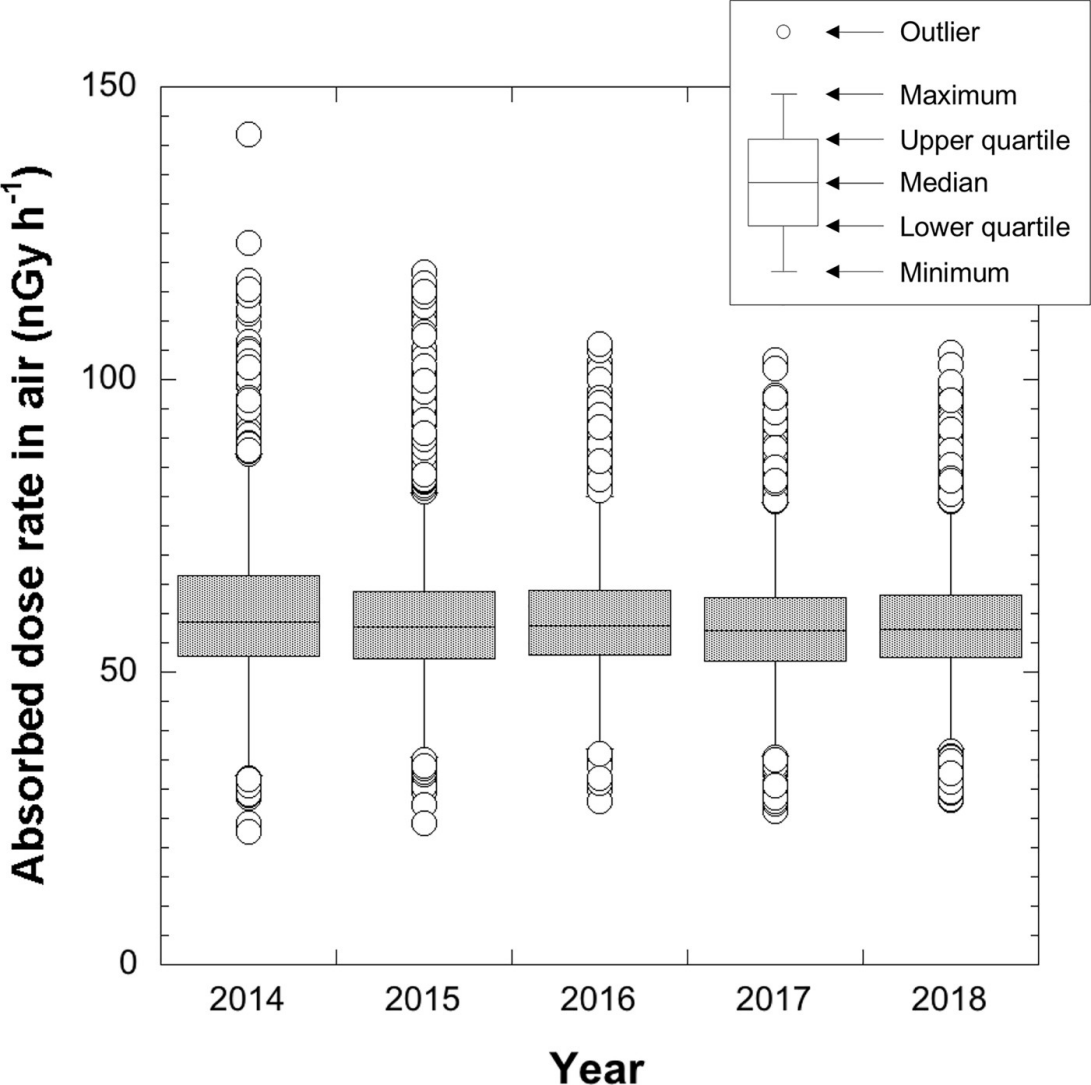
Calculated absorbed dose rates in air from natural and artificial radionuclides measured in 2014 [5]– 2018 in metropolitan Tokyo based on the measurements by the car-borne survey technique. The measurement was done on the same route (red line in Fig 2) using the same 3-in × 3-in NaI(Tl) scintillation spectrometer.

The absorbed dose rates in air (nGy h^-1^) outside the car 1 m above the ground surface were calculated using both *SF* and *DCF* (Eq 1). The changes of absorbed dose rates in air measured in 2014 [5], 2015, 2016, 2017 and 2018 are shown in Fig 3. The outliers were defined as: < lower quartile– 1.5 × distance from upper quartile to lower quartile (IQD) or > upper quartile + 1.5 × IQD (KaleidaGraph, Synergy Software, USA). The average absorbed dose rates in air (ranges) in metropolitan Tokyo were 60 ± 11 nGy h^-1^ (23–142 nGy h^-1^; *n* = 4,018) for 2014 [5], 59 ± 10 nGy h^-1^ (24–118 nGy h^-1^; *n* = 4,018) for 2015, 59 ± 9 nGy h^-1^ (28–106 nGy h^-1^; *n* = 4,346) for 2016, 58 ± 8 nGy h^-1^ (26–97 nGy h^-1^; *n* = 4,717) for 2017 and 59 ± 9 nGy h^-1^ (28–105 nGy h^-1^; *n* = 5,138) for 2018. The detailed absorbed dose rates in air in all municipalities in Tokyo are shown in S1 Table. According to the Tokyo Metropolitan Government, the average absorbed dose rate measured at 100 locations in June 2011 was 61 nGy h^-1^ (30–200 nGy h^-1^) [5]. The average absorbed dose rate in metropolitan Tokyo has not significantly changed in the past seven years but the number of high outliers (i.e., higher dose rates) has decreased yearly (Fig 3).

**Fig 4:**
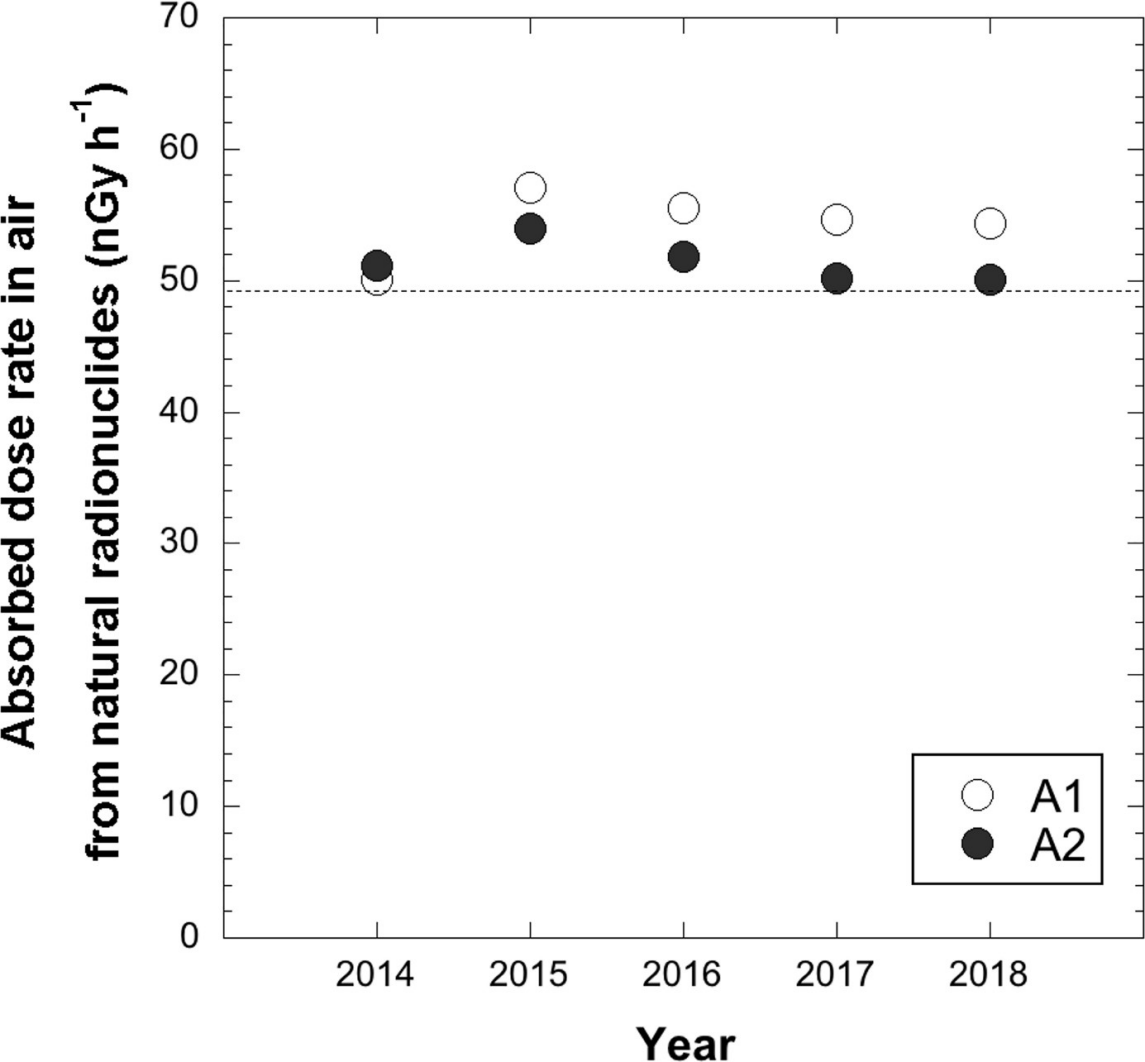
**Changes of absorbed dose rate in air from natural radionuclides in the eastern (A1) and western (A2) ends of Tokyo in 2014 [5]– 2018.** The gamma-ray pulse height distributions were measured outside the car for 10 min, at 61 locations (Fig 2). The gamma-ray pulse height distributions were then unfolded using the 22 × 22 response matrix method, and separated as natural radionuclides (K-40, U-238 series and Th-232 series).

Fig 4 shows average absorbed dose rate in air from natural radionuclides measured at 61 locations (Fig 2). Those dose rates were calculated using the 22 × 22 response matrix method [18]. Those dose rates were increased in the last 10 years compared to the measured dose rate in 2003 for Tokyo (49 ± 6 nGy h^-1^) [6], especially in A1 area. The construction of buildings and hotels has increased sharply since 2015 as Tokyo prepares to host the 2020 Olympics, and that has resulted in the increased dose rate because many natural radionuclides are contained in the building materials. In fact, the respective numbers of newly completed units for skyscrapers (i.e., more than twenty-story building) in metropolitan Tokyo in 2014 and 2015 were 5620 and 14738 according to the statistical data published by the Ministry of Land, Infrastructure, Transport and Tourism of Japan [27]. Additionally, absorbed dose rate in air from natural radionuclides changes depending on environmental conditions such as soil moisture and radon concentration. Thus, it is difficult to make a simple comparison on dose rates before and after the F1-NPP accident.

**Fig 5:**
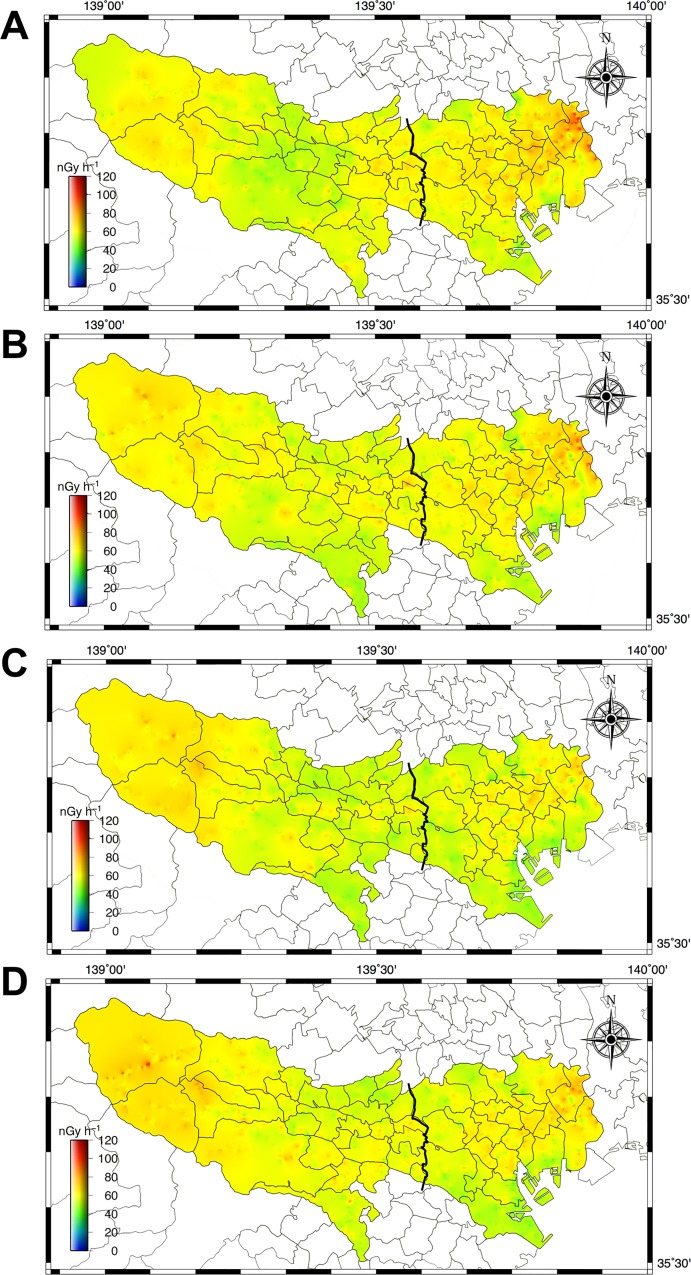
**The distribution maps of absorbed dose rates in air in metropolitan Tokyo measured in 2015 (A), 2016 (B), 2017 (C) and 2018 (D).** A minimum curvature algorithm was used for the data interpolation using the GMT [15]. Those maps were drawn using 4,018 data for 2015, 4,346 data for 2016, 4,717 data for 2017 and 5,138 data for 2018.

Fig 5 shows distribution maps of absorbed dose rate in air measured in 2015–2018 in metropolitan Tokyo. Those maps were drawn with the same magnification and altitude color gradation scale using GMT [15] and interpolated measured dose rates using a minimum curvature algorithm because the measurements of dose rate could not be performed at some areas. While absorbed dose rate in air in all municipalities in Tokyo are shown in S1 Table, there is limitation to the details that can be shown on the dose distribution map, especially in the mountain area at the western end of Tokyo. Additionally, changes of absorbed dose rate in air from natural radionuclides need to be considered to compare dose distribution maps as shown in Fig 4. In reported measurements of 2014 that were done in a car-borne survey on the same route and used the same NaI(Tl) scintillation spectrometer [5], higher dose rates exceeding 100 nGy h^-1^ were observed in Katsushika Ward (#22 in Fig 1) and Okutama Town (#53 in Fig 1), and their heterogeneous distributions were shown to be due to the presence of artificial radionuclides. The maps in Fig 5, however, showed the differences in dose rates yearly became smaller on the eastern and western ends of metropolitan Tokyo. The average absorbed dose rates in A1 and A2 in Fig 1 measured in 2014 were 60 ± 12 nGy h^-1^ (23–142 nGy h^-1^; *n* = 2,010) and 61 ± 10 nGy h^-1^ (32–102 nGy h^-1^; *n* = 2,255), respectively. After four years, those values measured in 2018 became 60 ± 9 nGy h^-1^ (28–105 nGy h^-1^; *n* = 2,216) and 58 ± 8 nGy h^-1^ (34–100 nGy h^-1^; *n* = 2,922), respectively, and those ranges of dose rates became smaller.

**Fig 6:**
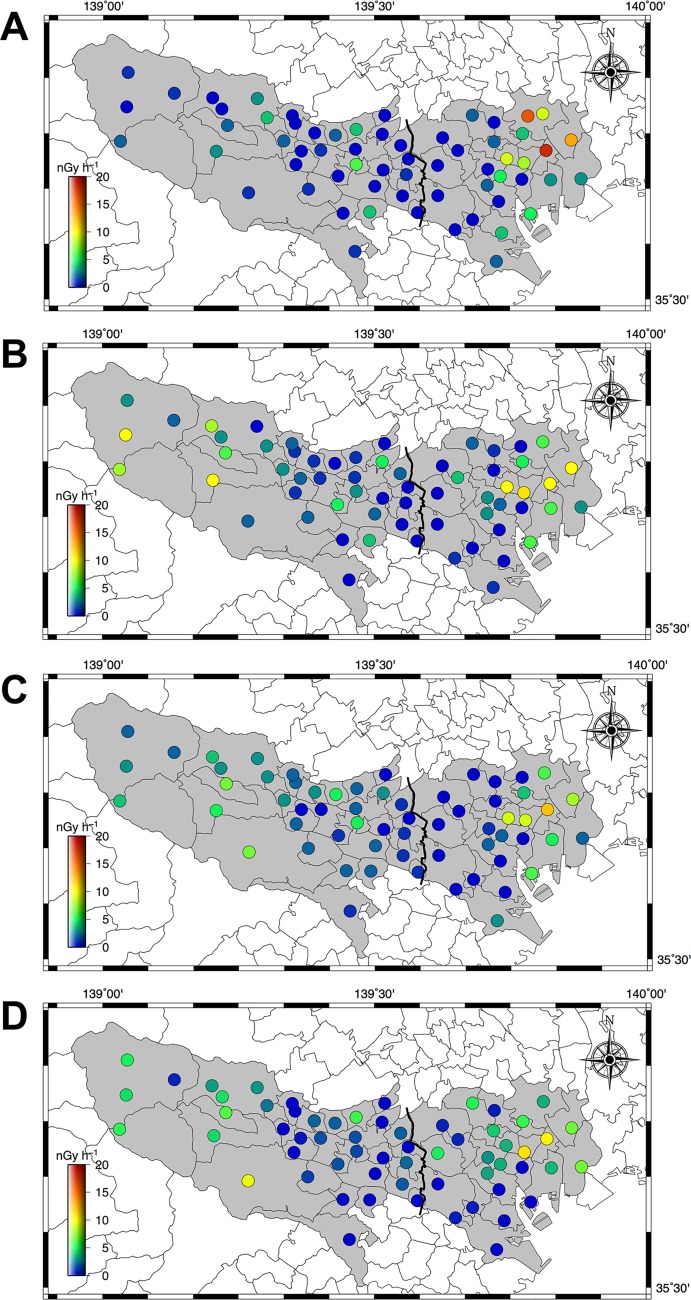
**The distribution maps of absorbed dose rates in air from artificial radionuclides in 2015 (A), 2016 (B), 2017 (C) and 2018 (D).** The gamma-ray pulse height distributions were measured for 10 min, at 61 locations (Fig 2). The gamma-ray pulse height distributions were then unfolded using the 22 × 22 response matrix method, and separated as artificial radionuclides (Cs-134 and Cs-137).

**Fig 7:**
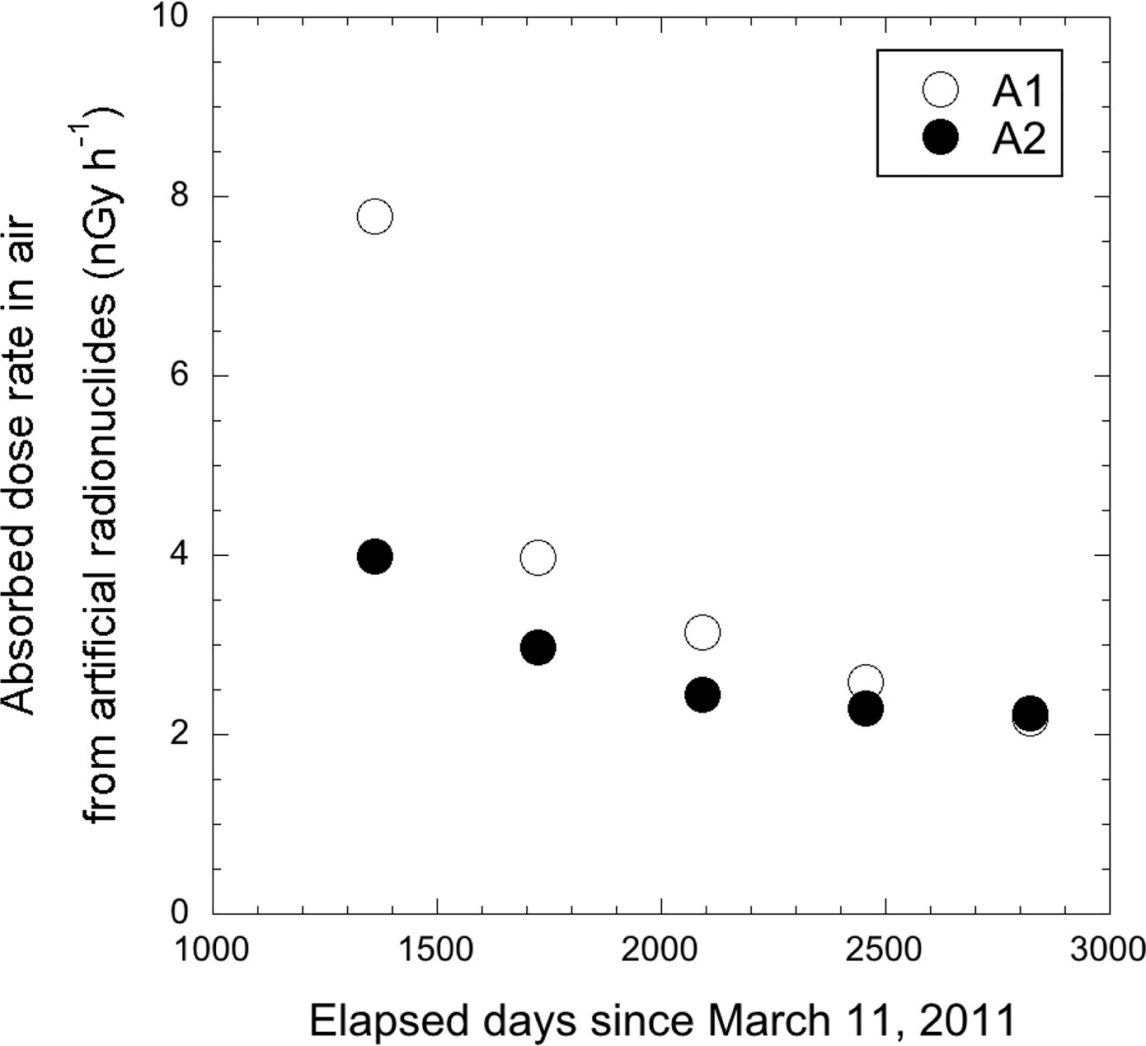
Changes of absorbed dose rate in air from artificial radionuclides in the eastern (A1) and western (A2) ends of Tokyo in 2014 [5]– 2018.

To allow more detailed discussion on changes of absorbed dose rates in air in metropolitan Tokyo, Fig 6 shows distribution maps of absorbed dose rates in air from two artificial radionuclides (Cs-134 + Cs-137). Those dose rates were calculated using the 22 × 22 response matrix method [18]. The average dose rates from artificial radionuclides for A1 and A2 areas are shown in Fig 7, and these values decreased yearly. The percent reductions for A1 area in the years 2014–2015, 2015–2016, 2016–2017 and 2017–2018 were 49%, 21%, 18% and 16%, and those percent reductions for A2 were 26%, 18%, 6% and 3%, respectively. The differences of percent reduction between A1 and A2 areas might be explained from the differences of the environment around measurement points. The percentages of road area [28] and green space [29] to total area of the administrative district are 16.5% and 3.8–23.1% for A1 area whereas those for A2 area are 6.7% and 30–97%. The deposited radionuclides on sealed surfaces such as asphalt or concrete pavements can be easily washed away by rainfall compared to bare ground or lichen-covered areas [5, 17, 30, 31]. Thus, a different reduction of dose rate between A1 and A2 areas was observed that was related to the two environment extremes. Additionally, the reduction ratios in 2014–2015 for both areas were the highest compared to other time periods (i.e., after 2016), and percent reductions then became lower yearly. Thus, it seems that the reduction of absorbed dose rate in air in A1 area was related to ecological effects such as weathering that occurred during the early term after the F1-NPP accident. These findings corresponded to those of the previous report for the F1-NPP accident [32]. In the Chernobyl accident, reduction ratios of Cs-137 contamination on street pavements were faster than that on a reference surface (a cut lawn) [33], and the same tendency was observed after the Fukushima accident as well [34].

**Fig 8:**
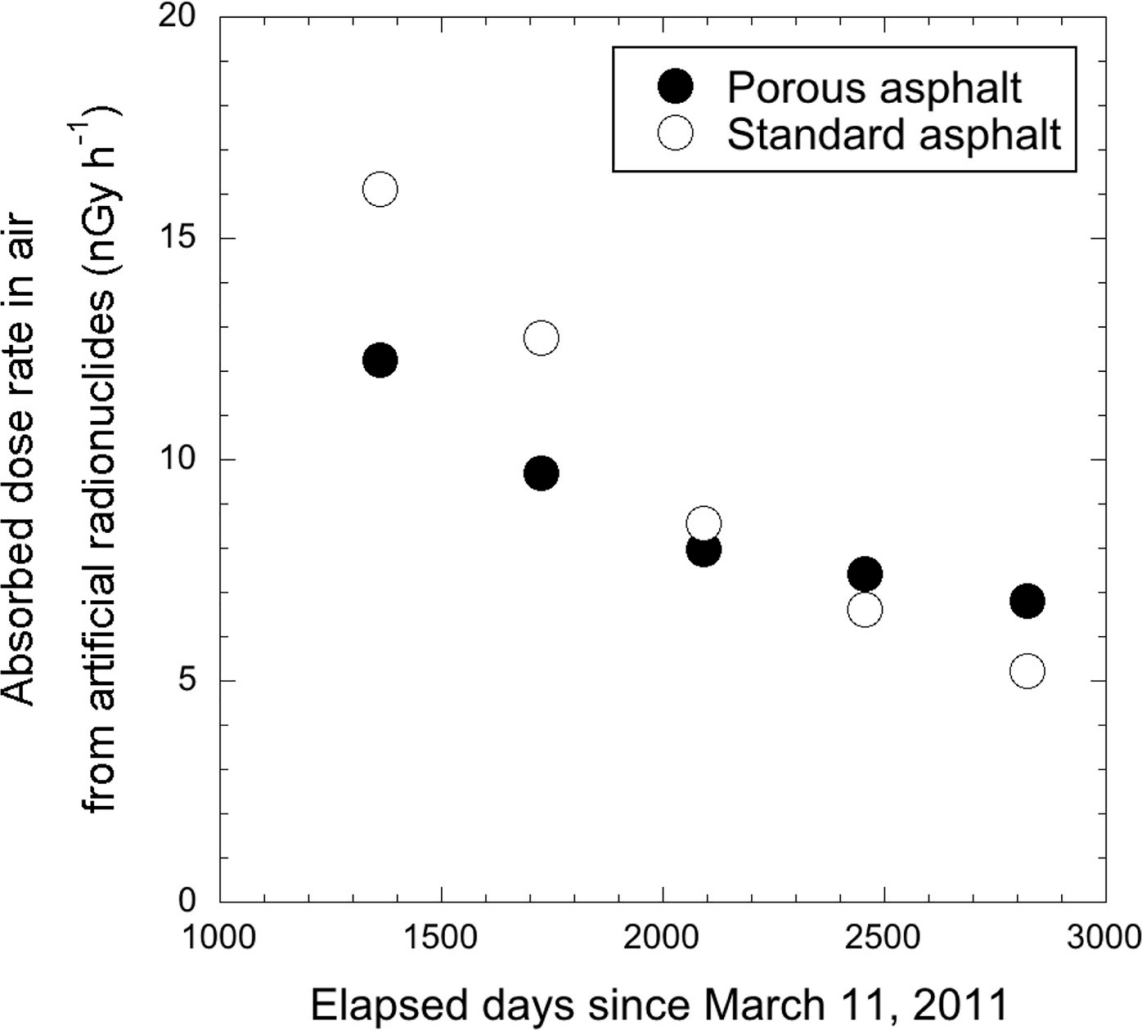
Changes of absorbed dose rate in air from artificial radionuclides measured at 1 m above the surface of porous and standard asphalt surfaces. The gamma-ray pulse height distributions were measured 1 m above the surface of porous (*n* = 3) and standard asphalt (*n* = 5) materials for 10 min. The gamma-ray pulse height distributions were then unfolded using the 22 × 22 response matrix method, and dose rates were calculated for the artificial radionuclides.

**Fig 9:**
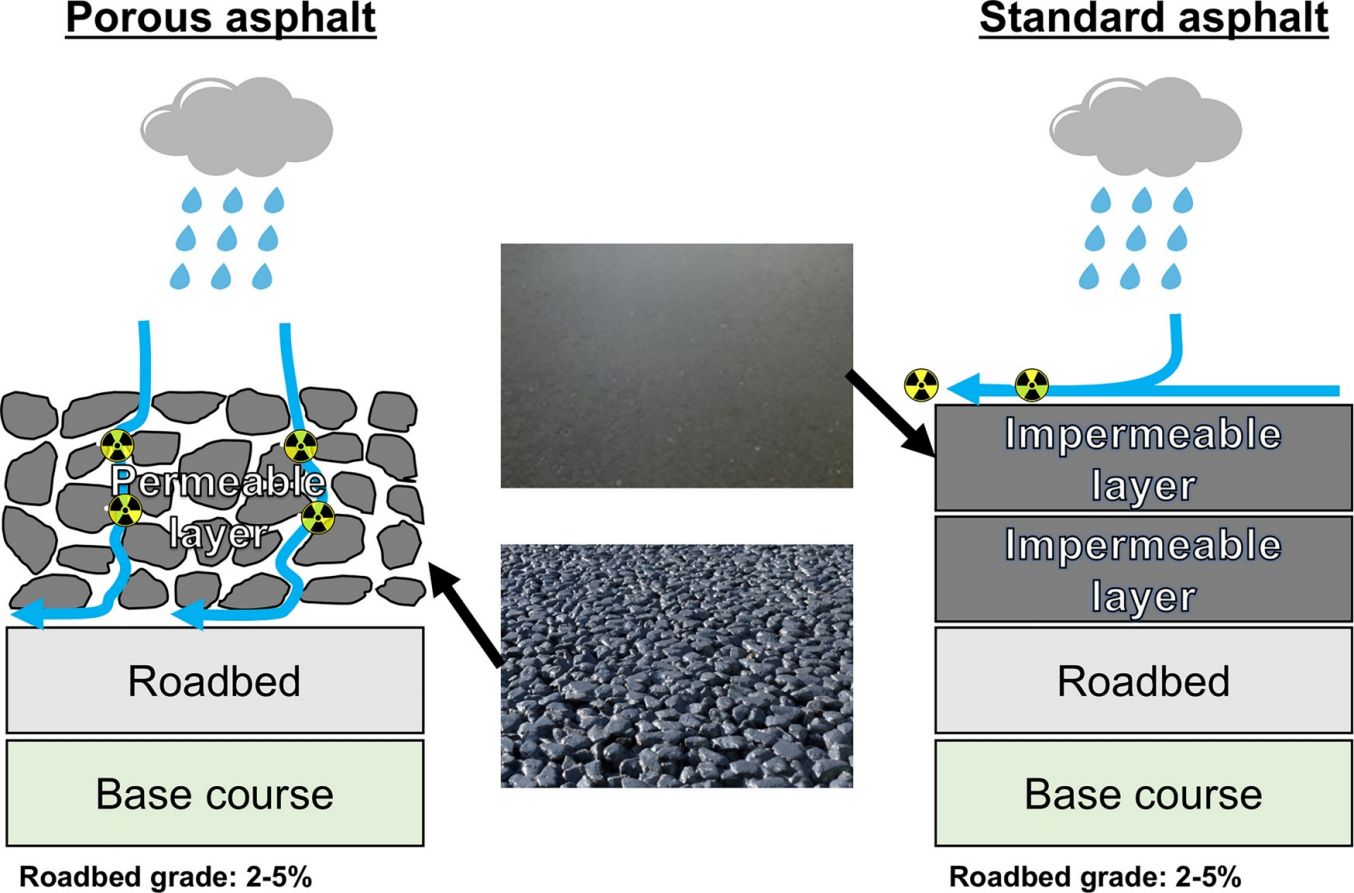
The structures of porous and standard asphalt materials.

For a more detailed evaluation, the reduction ratio of absorbed dose rate in air related with type of asphalt was analyzed using measured data from the Joto district (#6 –#8 and #21 –#23 in Fig 1) which is a highly contaminated area in metropolitan Tokyo compared to that in other nearby wards. Fig 8 shows the transition of absorbed dose rate in air from artificial radionuclides measured at 1 m above porous asphalt (*n* = 3, #21 –#22 in Fig 1) and standard asphalt (*n* = 5, #6 –#8 and #23 in Fig 1) surfaces. The percent reductions of dose rate in the years 2014–2015, 2015–2016, 2016–2017 and 2017–2018 were 21%, 18%, 7% and 7% for porous asphalt, and those ratios for standard asphalt were 21%, 37%, 18% and 21%, respectively. Therefore, the reduction of dose rates measured on standard asphalt was occurring faster than that on porous asphalt. This can be explained from the structure difference of both asphalt types. The coarse aggregate diameters of the two are different (Fig 9). The porous asphalt material consists of coarse aggregates with a diameter of more than 2.36 mm, and the drainage function is high, resulting in its wide use recently for highways and main roads. However, it can be quickly clogged by dust depending on the amount of traffic, and deposited radiocesium that became attached to the dust particles has the property of binding strongly with the dust particles and not being easily washed away by rainfall. [31]. On the other hand, standard asphalt is low porosity asphalt consisting of fine aggregates having diameters of 0.075–2.36 mm and it is utilized for local roads and public parking areas. This type of asphalt has a water repellency effect and the deposited radiocesium and dust particles are easily washed out by rainfall compared to high porosity asphalt. Thus, the dose rate measured on standard asphalt decreased more quickly compared with that on porous asphalt. When the changing dose rates are locally evaluated, it seems that the dose rates would not be homogeneously decreased due to the asphalt type dependency.

### Uncertainties on car-borne survey technique and fixed-point observation

The relative standard uncertainties for the shielding factor, dose conversion factor, traceability of the dose rate (calculated by Pony Industry Co., Ltd., Osaka, Japan) and the dose calculation procedure by the response matrix method (calculated by EMF Japan Co., Osaka, Japan) were obtained as 5.3%, 6.2%, 4.1% (*k* = 2) and 5.0%, respectively. Additionally, the relative standard uncertainties of one-time measurement (30 s) and fixed-point observation (10 min) were calculated to be 3.0% and 0.2%, respectively. The combined relative standard uncertainties of the car-bone survey and fixed-point observation in the present study were calculated to be 10.8% and 10.4%, respectively. However, the real combined relative standard uncertainty would be estimated as more than 10.4% because the uncertainty for the separation of absorbed dose rate in air from natural and artificial radionuclides could not be considered in this study.

## Conclusion

Car-borne surveys with a NaI(Tl) scintillation spectrometer were carried out for metropolitan Tokyo during 2015–2018 and the changes of absorbed dose rate in air related to the radionuclide releases from the F1-NPP accident were discussed, including data measured in 2011 and 2014. While absorbed dose rates in air in higher contaminated areas exceeding 70 nGy h^-1^ have been decreasing yearly, the average absorbed dose rate in air from natural and artificial radionuclides for the whole metropolitan Tokyo area was not significantly changed during the seven years from 2011 (61 ± 24 nGy h^-1^) to 2018 (59 ± 9 nGy h^-1^). The decreasing rate of absorbed dose rate in air from artificial radionuclides in the eastern end of Tokyo which is mainly covered by asphalt was higher than that measured in the western end of Tokyo which is mainly covered by forests. The decreased dose rates strongly depended on type of asphalt pavement, and the reduction ratio of dose rate measured at 1 m above the porous asphalt surface was longer than that of standard asphalt.

## Supporting information

S1 Table Absorbed dose rate in air in all municipalities in Tokyo measured in 2014, 2015, 2016, 2017 and 2018. (DOCX)

## References

[1] Saito K, Tanihata I, Fujiwara M, Saito T, Shimoura S, Otsuka T, et al Detailed deposition density maps constructed by large-scale soil sampling for gamma-ray emitting radioactive nuclides from the Fukushima Dai-ichi Nuclear Power Plant accident. J Environ Radioact. 2015;139:308–19. 10.1016/j.jenvrad.2014.02.014 24703526

[2] United Nations Scientific Committee on the Effects of Atomic Radiation. Developments since the 2013 UNSCEAR Report on the levels and effects of radiation exposure due to the nuclear accident following the great east-Japan earthquake and tsunami, A 2017 White Paper to guide the Scientific Committee's future programme of work. 2017.

[3] Kinoshita N, Sueki K, Sasa K, Kitagawa J, Ikarashi S, Nishimura T, et al Assessment of individual radionuclide distributions from the Fukushima nuclear accident covering central-east Japan. P Natl Acad Sci USA. 2011;108(49):19526–9. 10.1073/pnas.1111724108 22084070 PMC3241760

[4] Morino Y, Ohara T, Nishizawa M. Atmospheric behavior, deposition, and budget of radioactive materials from the Fukushima Daiichi nuclear power plant in March 2011. Geophysical Research Letters. 2011;38(7):L00G11 10.1029/2011GL048689

[5] Inoue K, Tsuruoka H, Van Le T, Arai M, Saito K, Fukushi M. Impact on ambient dose rate in metropolitan Tokyo from the Fukushima Daiichi Nuclear Power Plant accident. J Environ Radioact. 2016;158–159:1–8. 10.1016/j.jenvrad.2016.03.022 27055250

[6] Inoue K, Hosoda M, Fukushi M, Furukawa M, Tokonami S. Absorbed dose rate in air in metropolitan Tokyo before the Fukushima Daiichi Nuclear Power Plant accident. Radiat Prot Dosimetry. 2015;(1–3):231–4. 10.1093/rpd/ncv251 25944962

[7] Inoue K, Hosoda M, Shiroma Y, Furukawa M, Fukushi M, Iwaoka K, et al Changes of ambient gamma-ray dose rate in Katsushika Ward, metropolitan Tokyo before and after the Fukushima Daiichi Nuclear Power Plant accident. J Radioanal Nucl Chem. 2015;303(3):2159–63. 10.1007/s10967-014-3669-x

[8] Nuclear Regulation Authority. Extension site of distribution map of radiation dose, etc. 2011. Available from: http://radioactivity.nsr.go.jp/en/. Accessed 25 June 2019.

[9] Andersson I, Lönsjö H, Rosén K. Long-term studies on transfer of ^137^Cs from soil to vegetation and to grazing lambs in a mountain area in Northern Sweden. J Environ Radioact. 2001;52(1):45–66. 10.1016/S0265-931X(00)00102-8 11202685

[10] Pröhl G, Ehlken S, Fiedler I, Kirchner G, Klemt E, Zibold G. Ecological half-lives of ^90^Sr and ^137^Cs in terrestrial and aquatic ecosystems. J Environ Radioact. 2006;91(1):41–72. 10.1016/j.jenvrad.2006.08.004 17007973

[11] Maedera F, Inoue K, Sugino M, Sano R, Furue M, Shimizu H, et al Natural Variation of Ambient Dose Rate in the Air of Izu-Oshima Island after the Fukushima Daiichi Nuclear Power Plant Accident. Radiat Prot Dosimetry. 2006;168(4):561–565. 10.1093/rpd/ncv370 26246583

[12] Isaksson M, Erlandsson B, Mattsson S. A 10-year study of the ^137^Cs distribution in soil and a comparison of Cs soil inventory with precipitation-determined deposition. J Environ Radioact. 2001;55(1):47–59. 10.1016/S0265-931X(00)00186-7 11381552

[13] Saegusa J, Yoda T, Murakami M, Takeishi M. Analysis of ambient-dose-rate trends in Fukushima Ecological half-life, effect of snow covering. Kankyo Hoshano Josen Gakkai-Shi. 2017;5(2):79–93. (Japanese with English abstract)

[14] Tokyo Metropolitan Institute of Public Health. Measurement results of environmental radiation levels in Tokyo 2015. Available from: http://monitoring.tokyo-eiken.go.jp/en/index.html. Accessed 25 June 2019.

[15] Wessel P. Free software helps map and display data. EOS, Trans Am Geophys Union. 1991;72:441–8. 10.1029/90EO00319

[16] Geospatial Information Authority of Japan. Elevation–Global version. Available from: https://globalmaps.github.io/el.html. Accessed 25 June 2019.

[17] Hosoda M, Tokonami S, Sorimachi A, Monzen S, Osanai M, Yamada M, et al The time variation of dose rate artificially increased by the Fukushima nuclear crisis. Sci Rep. 2011;1:87 10.1038/srep00087 22355606 PMC3216573

[18] Minato S. Diagonal Elements Fitting Technique to Improve Response Matrixes for Environmental Gamma Ray Spectrum Unfolding. Radioisot. 2001;50(10):463–71. 10.3769/radioisotopes.50.10_463

[19] Hosoda M, Tokonami S, Omori Y, Sahoo SK, Akiba S, Sorimachi A, et al Estimation of external dose by car-borne survey in Kerala, India. PLoS One. 2015;10(4):e0124433 10.1371/journal.pone.0124433 25885680 PMC4401755

[20] Matsuda H, Furukawa S, Kaminishi T, Minato S, A new method for evaluating weak leakage gamma-ray dose using a 3”φ × 3” NaI(Tl) scintillation spectrometer (I) Principle of background estimation method. Rep Gov Ind Res Inst. 1982;31:132–146 (in Japanese).

[21] Le TV, Inoue K, Tsuruoka H, Fujisawa M, Arai M, Nguyen LDH, et al Effective Dose due to Terrestrial Gamma Radiation Estimated in Southern Vietnam by Car-Borne Survey Technique. Radiat Prot Dosimetry. 2018;179(1):18–25. 10.1093/rpd/ncx185 29036482

[22] Inoue K, Arai M, Fujisawa M, Saito K, Fukushi M. Detailed Distribution Map of Absorbed Dose Rate in Air in Tokatsu Area of Chiba Prefecture, Japan, Constructed by Car-Borne Survey 4 Years after the Fukushima Daiichi Nuclear Power Plant Accident. PLoS One. 2017;12(1):e0171100 10.1371/journal.pone.0171100 28129382 PMC5271352

[23] Hosoda M, Inoue K, Oka M, Omori Y, Iwaoka K, Tokonami S. Environmental Radiation Monitoring and External Dose Estimation in Aomori Prefecture after the Fukushima Daiichi Nuclear Power Plant Accident. Jpn J Health Phys. 2016;51(1):41–50. 10.5453/jhps.51.41

[24] Inoue K, Tsuruoka H, Le Van T, Fukushi M. Contribution ratios of natural radionuclides to ambient dose rate in air after the Fukushima Daiichi Nuclear Power Plant accident. J Radioanal Nucl Chem. 2015:1–6. 10.1007/s10967-015-4164-8

[25] Inoue K, Hosoda M, Sugino M, Simizu H, Akimoto A, Hori K, et al Environmental radiation at Izu-Oshima after the Fukushima Daiichi nuclear power plant accident. Radiat Prot Dosimetry. 2012;152(1–3):234–7. 10.1093/rpd/ncs228 22927656

[26] Shimo M, Minato S, Sugino M. A Survey of Environmental Radiation in Aichi, Gifu and Mie Prefectures. J Nucl Sci Technol. 1999;41(9):954–964. 10.3327/jaesj.41.954 (Japanese with English abstract)

[27] Ministry of Land, Infrastructure, Transport and Tourism. Housing and economic data. http://www.mlit.go.jp/statistics/details/t-jutaku-2_tk_000002.html. (in Japanese) Accessed 4 September 2019.

[28] Bureau of Construction, Tokyo Metropolitan Government. Available from: http://www.kensetsu.metro.tokyo.jp/english/index.html. Accessed 25 June 2019.

[29] Institute for Community and Life Resources. Available from: http://www.chiikiseikatsu.org/databook2014/databook2014tnk.pdf. Accessed 25 June 2019. (Japanese)

[30] Mueck K, Steger F. Wash-Off Effects in Urban Areas. Radiat Prot Dosimetry. 1991;37(1):189–94. 10.1093/oxfordjournals.rpd.a081051

[31] Roed J, Andersson K, Barkovsly A, Fogh C, Mishine A, Olsen S, et al Mechanical decontamination tests in areas affected by the Chernobyl accident. 8 1998 http://www.iaea.org/inis/collection/NCLCollectionStore/_Public/30/004/30004765.pdf. Accessed 25 June 2019.

[32] Tsubokura M, Murakami M, Nomura S, Morita T, Nishikawa Y, Leppold C, Kato S, Kami M. Individual external doses below the lowest reference level of 1 mSv per year five years after the 2011 Fukushima nuclear accident among all children in Soma City, Fukushima: A retrospective observational study. PLoS One. 2017;12(2):e0172305 10.1371/journal.pone.0172305 28235009 PMC5325236

[33] Andersson KG, Roed J, Fogh CL. Weathering of radiocaesium contamination on urban streets, walls and roofs. J Environ Radioact. 2002;62(1):49–60. 10.1016/S0265-931X(01)00150-3 12141607

[34] Yoshimura K, Saito K, Fujiwara K. Distribution of ^137^Cs on components in urban area four years after the Fukushima Dai-ichi Nuclear Power Plant accident. J Environ Radioact. 2017;178–179:48–54. 10.1016/j.jenvrad.2017.07.021 28778008

