# Supplementary material for: Changes of absorbed dose rate in air in metropolitan Tokyo relating to radiocesium released from the Fukushima Daiichi Nuclear Power Plant accident: Results of a five-year study
Source: PLoS One. 2019 Oct 24;14(10):e0224449. doi: 10.1371/journal.pone.0224449 (PMC6812831; doi:10.1371/journal.pone.0224449)
Supplement: S1 Table — (DOCX) [file pone.0224449.s001.docx]

**S1 Table** Absorbed dose rate in air in all municipalities in Tokyo measured in 2014, 2015, 2016, 2017 and 2018.

| No.^a^ | Municipality | Absorbed dose rate in air (nGy h^-1^) | | | | | | | | | | | | | | | | | | | |
| --- | --- | --- | --- | --- | --- | --- | --- | --- | --- | --- | --- | --- | --- | --- | --- | --- | --- | --- | --- | --- | --- |
|  |  | 2014^b^ | | | | 2015 | | | | 2016 | | | | 2017 | | | | 2018 | | | |
|  |  | Mean | Range | | | Mean | Range | | | Mean | Range | | | Mean | Range | | | Mean | Range | | |
| 1 | Chiyoda Ward | 62 | 23 | – | 80 | 64 | 44 | – | 93 | 61 | 44 | – | 84 | 59 | 41 | – | 76 | 63 | 46 | – | 81 |
| 2 | Chuoh Ward | 66 | 49 | – | 86 | 68 | 52 | – | 105 | 66 | 47 | – | 88 | 65 | 52 | – | 75 | 66 | 50 | – | 89 |
| 3 | Minato Ward | 56 | 23 | – | 75 | 64 | 42 | – | 90 | 62 | 46 | – | 84 | 57 | 26 | – | 85 | 62 | 34 | – | 79 |
| 4 | Shinjuku Ward | 64 | 48 | – | 90 | 64 | 40 | – | 88 | 62 | 42 | – | 78 | 60 | 50 | – | 80 | 61 | 44 | – | 79 |
| 5 | Bunkyo Ward | 61 | 47 | – | 81 | 65 | 48 | – | 79 | 64 | 44 | – | 78 | 60 | 46 | – | 73 | 60 | 47 | – | 75 |
| 6 | Taito Ward | 69 | 44 | – | 86 | 72 | 49 | – | 86 | 68 | 52 | – | 81 | 65 | 34 | – | 85 | 62 | 47 | – | 76 |
| 7 | Sumida Ward | 65 | 32 | – | 93 | 67 | 42 | – | 90 | 64 | 39 | – | 84 | 61 | 42 | – | 81 | 65 | 48 | – | 87 |
| 8 | Koto Ward | 53 | 29 | – | 89 | 57 | 32 | – | 86 | 53 | 30 | – | 84 | 53 | 27 | – | 77 | 56 | 33 | – | 80 |
| 9 | Shinagawa Ward | 52 | 37 | – | 73 | 56 | 24 | – | 76 | 60 | 42 | – | 75 | 52 | 35 | – | 72 | 50 | 31 | – | 67 |
| 10 | Meguro Ward | 53 | 43 | – | 64 | 57 | 47 | – | 65 | 56 | 48 | – | 69 | 57 | 47 | – | 68 | 56 | 45 | – | 65 |
| 11 | Ohta Ward | 52 | 29 | – | 65 | 54 | 37 | – | 72 | 54 | 31 | – | 69 | 52 | 36 | – | 71 | 51 | 32 | – | 70 |
| 12 | Setagaya Ward | 55 | 38 | – | 77 | 59 | 39 | – | 73 | 59 | 44 | – | 72 | 57 | 41 | – | 76 | 56 | 38 | – | 73 |
| 13 | Shibuya Ward | 64 | 47 | – | 84 | 64 | 48 | – | 83 | 64 | 31 | – | 78 | 61 | 42 | – | 77 | 62 | 44 | – | 83 |
| 14 | Nakano Ward | 54 | 43 | – | 67 | 62 | 49 | – | 72 | 64 | 51 | – | 74 | 56 | 38 | – | 69 | 59 | 38 | – | 74 |
| 15 | Suginami Ward | 59 | 43 | – | 72 | 60 | 40 | – | 77 | 60 | 40 | – | 73 | 59 | 47 | – | 72 | 57 | 45 | – | 76 |
| 16 | Toshima Ward | 61 | 46 | – | 83 | 64 | 45 | – | 81 | 59 | 49 | – | 76 | 62 | 49 | – | 73 | 62 | 42 | – | 80 |
| 17 | Kita Ward | 58 | 32 | – | 83 | 64 | 42 | – | 82 | 59 | 38 | – | 81 | 57 | 34 | – | 72 | 58 | 34 | – | 78 |
| 18 | Arakawa Ward | 69 | 45 | – | 94 | 73 | 54 | – | 89 | 60 | 46 | – | 76 | 58 | 36 | – | 78 | 57 | 32 | – | 82 |
| 19 | Itabashi Ward | 57 | 43 | – | 83 | 61 | 35 | – | 76 | 59 | 44 | – | 73 | 57 | 45 | – | 75 | 56 | 39 | – | 73 |
| 20 | Nerima Ward | 56 | 40 | – | 82 | 60 | 36 | – | 76 | 57 | 37 | – | 78 | 56 | 40 | – | 76 | 55 | 40 | – | 73 |
| 21 | Adachi Ward | 59 | 31 | – | 95 | 61 | 27 | – | 96 | 61 | 34 | – | 88 | 57 | 31 | – | 82 | 56 | 28 | – | 84 |
| 22 | Katsushika Ward | 73 | 31 | – | 142 | 76 | 36 | – | 118 | 67 | 28 | – | 105 | 63 | 33 | – | 96 | 67 | 32 | – | 105 |
| 23 | Edogawa Ward | 64 | 36 | – | 115 | 64 | 34 | – | 115 | 63 | 36 | – | 106 | 56 | 30 | – | 88 | 66 | 40 | – | 98 |
| 24 | Hachiohji City | 64 | 46 | – | 88 | 54 | 39 | – | 84 | 58 | 32 | – | 86 | 58 | 41 | – | 88 | 62 | 46 | – | 100 |
| 25 | Tachikawa City | 55 | 45 | – | 75 | 51 | 42 | – | 65 | 57 | 46 | – | 71 | 58 | 45 | – | 79 | 57 | 43 | – | 75 |
| 26 | Musashino City | 58 | 48 | – | 73 | 59 | 47 | – | 71 | 63 | 49 | – | 74 | 57 | 43 | – | 71 | 64 | 47 | – | 83 |
| 27 | Mitaka City | 55 | 43 | – | 66 | 62 | 52 | – | 75 | 59 | 36 | – | 96 | 56 | 40 | – | 87 | 60 | 46 | – | 76 |
| 28 | Oume City | 67 | 48 | – | 96 | 56 | 43 | – | 91 | 57 | 43 | – | 76 | 60 | 43 | – | 94 | 59 | 38 | – | 93 |
| 29 | Fuchu City | 57 | 35 | – | 74 | 60 | 40 | – | 83 | 61 | 40 | – | 84 | 57 | 47 | – | 89 | 58 | 40 | – | 83 |
| 30 | Akishima City | 55 | 46 | – | 77 | 53 | 40 | – | 69 | 57 | 48 | – | 77 | 58 | 39 | – | 82 | 55 | 42 | – | 70 |
| 31 | Chofu City | 56 | 46 | – | 68 | 60 | 46 | – | 73 | 58 | 46 | – | 71 | 54 | 41 | – | 62 | 57 | 39 | – | 71 |
| 32 | Machida City | 54 | 42 | – | 77 | 55 | 40 | – | 108 | 52 | 40 | – | 77 | 53 | 28 | – | 97 | 55 | 40 | – | 98 |
| 33 | Koganei City | 60 | 50 | – | 73 | 64 | 51 | – | 83 | 61 | 48 | – | 78 | 57 | 42 | – | 78 | 60 | 47 | – | 73 |
| 34 | Kodaira City | 56 | 46 | – | 69 | 58 | 49 | – | 73 | 56 | 44 | – | 69 | 54 | 46 | – | 66 | 54 | 42 | – | 67 |
| 35 | Hino City | 66 | 55 | – | 78 | 54 | 44 | – | 90 | 56 | 44 | – | 70 | 57 | 43 | – | 73 | 60 | 47 | – | 77 |
| 36 | Higashimurayama City | 54 | 42 | – | 64 | 59 | 48 | – | 78 | 56 | 42 | – | 72 | 55 | 40 | – | 69 | 54 | 43 | – | 71 |
| 37 | Kokubunji City | 56 | 45 | – | 74 | 54 | 43 | – | 73 | 57 | 44 | – | 69 | 56 | 40 | – | 72 | 57 | 43 | – | 78 |
| 38 | Kunitachi City | 59 | 48 | – | 71 | 55 | 44 | – | 73 | 62 | 49 | – | 81 | 55 | 44 | – | 67 | 59 | 47 | – | 74 |
| 39 | Nishitokyo City | 58 | 46 | – | 74 | 60 | 48 | – | 75 | 58 | 43 | – | 77 | 56 | 46 | – | 70 | 57 | 49 | – | 68 |
| 40 | Fussa City | 57 | 47 | – | 69 | 53 | 44 | – | 63 | 60 | 50 | – | 69 | 57 | 35 | – | 79 | 58 | 49 | – | 75 |
| 41 | Komae City | 57 | 42 | – | 71 | 59 | 48 | – | 70 | 60 | 46 | – | 67 | 56 | 40 | – | 69 | 57 | 45 | – | 72 |
| 42 | Higashiyamato City | 54 | 42 | – | 64 | 58 | 49 | – | 69 | 56 | 47 | – | 64 | 52 | 41 | – | 60 | 54 | 46 | – | 67 |
| 43 | Kiyose City | 55 | 47 | – | 65 | 61 | 45 | – | 75 | 58 | 44 | – | 76 | 57 | 45 | – | 72 | 54 | 42 | – | 68 |
| 44 | Higashikurume City | 56 | 44 | – | 68 | 59 | 48 | – | 77 | 55 | 44 | – | 73 | 55 | 38 | – | 73 | 50 | 40 | – | 64 |
| 45 | Musashimurayama City | 53 | 42 | – | 67 | 56 | 46 | – | 64 | 53 | 44 | – | 63 | 52 | 42 | – | 71 | 53 | 46 | – | 65 |
| 46 | Tama City | 56 | 41 | – | 69 | 56 | 38 | – | 72 | 55 | 43 | – | 70 | 54 | 44 | – | 70 | 60 | 44 | – | 84 |
| 47 | Inagi City | 55 | 32 | – | 76 | 60 | 52 | – | 81 | 58 | 44 | – | 88 | 53 | 43 | – | 60 | 59 | 48 | – | 79 |
| 48 | Hamura City | 57 | 41 | – | 73 | 55 | 45 | – | 65 | 61 | 52 | – | 77 | 56 | 43 | – | 67 | 59 | 50 | – | 69 |
| 49 | Akiruno City | 68 | 46 | – | 87 | 59 | 43 | – | 88 | 61 | 44 | – | 92 | 62 | 45 | – | 84 | 62 | 34 | – | 92 |
| 50 | Mizuho Town | 53 | 45 | – | 70 | 52 | 40 | – | 65 | 53 | 45 | – | 65 | 55 | 42 | – | 68 | 56 | 46 | – | 73 |
| 51 | Hinode Town | 65 | 56 | – | 76 | 54 | 46 | – | 60 | 58 | 48 | – | 67 | 60 | 45 | – | 68 | 61 | 52 | – | 72 |
| 52 | Hinohara Village | 73 | 37 | – | 95 | 63 | 53 | – | 83 | 62 | 53 | – | 74 | 64 | 49 | – | 80 | 66 | 50 | – | 86 |
| 53 | Okutama Town | 74 | 59 | – | 102 | 61 | 34 | – | 77 | 65 | 41 | – | 94 | 65 | 45 | – | 87 | 66 | 45 | – | 124 |

^a^ The numbers refer to the designations in Fig 1.

^b^ Inoue et al. [5]
